# Spatially selective responses to Kanizsa and occlusion stimuli in human visual cortex

**DOI:** 10.1038/s41598-017-19121-z

**Published:** 2018-01-12

**Authors:** Benjamin de Haas, Dietrich Samuel Schwarzkopf

**Affiliations:** 1 UCL Experimental Psychology, 26 Bedford Way, London, UK; 2 UCL Institute of Cognitive Neuroscience, 17 Queen Square, London, UK; 3 JLU Department of Psychology, Otto-Behagel-Str. 10 F, Giessen, Germany; 4 School of Optometry and Vision Science, University of Auckland, 85 Park Road, Auckland, New Zealand

## Abstract

Early visual cortex responds to illusory contours in which abutting lines or collinear edges imply the presence of an occluding surface, as well as to occluded parts of an object. Here we used functional magnetic resonance imaging (fMRI) and population receptive field (pRF) analysis to map retinotopic responses in early visual cortex using bar stimuli defined by illusory contours, occluded parts of a bar, or subtle luminance contrast. All conditions produced retinotopic responses in early visual field maps even though signal-to-noise ratios were very low. We found that signal-to-noise ratios and coherence with independent high-contrast mapping data increased from V1 to V2 to V3. Moreover, we found no differences of signal-to-noise ratios or pRF sizes between the low-contrast luminance and illusion conditions. We propose that all three conditions mapped spatial attention to the bar location rather than activations specifically related to illusory contours or occlusion.

## Introduction

**Figure 1:**
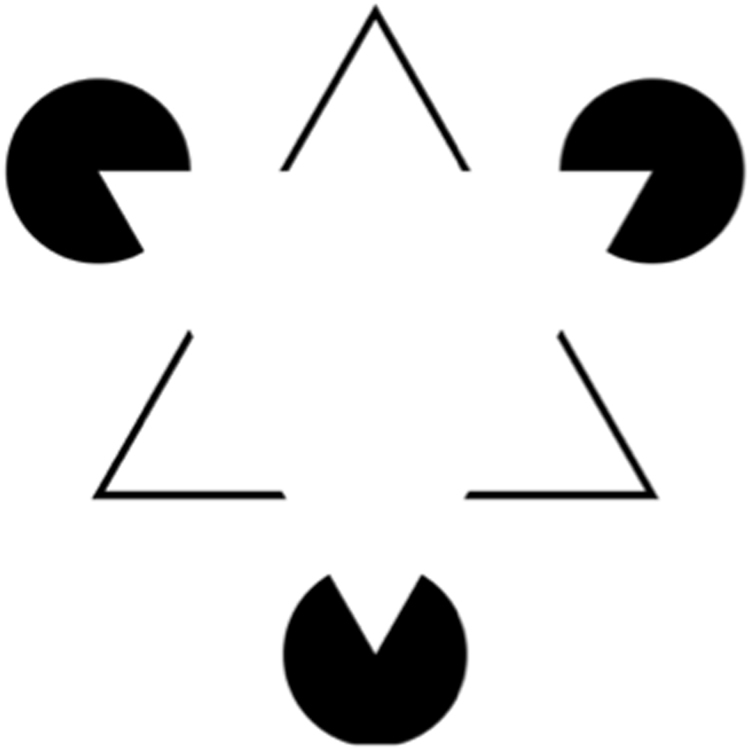
The Kanizsa triangle illustrates two forms of filling in: illusory contours and amodal completion. Illusory contours: A foreground white triangle results from the alignment of black Pacmen and abutting ‘V’ shaped segments. This region is perceived as brighter than the background and thus an illusory edge is perceived. Amodal completion: The ‘V’ shaped segments are perceived as a complete but occluded black triangle outline behind the white triangle^44^.

To interpret a visual scene an observer has to differentiate between foreground and background, for instance by detecting the edges separating one triangle overlapping another. However, when two objects are identically coloured, their relational contours must be inferred. An illusory contour occurs when the observer subjectively perceives an edge or surface defined by a difference in brightness, even though there is no luminance contrast edge. Conversely, in occlusion or “amodal completion”, one object is interpreted as being behind another object even though only fragments of it are visible (Fig. 1). These processes demonstrate that human visual perception is not merely a direct representation of the light patterns received by the retina but an inferential process.

Several studies reported that both illusory contours and occlusion produce neural activity in early visual cortex^1–8^. It remains controversial whether illusory contours and occlusion share a common neural mechanism and whether they reflect a bottom-up or top-down process. The perceptual differences between illusory contours and occlusion suggest that their neural activation should also be dissociable in some brain regions. Further evidence for different neural mechanisms stems from the analysis of two callosotomy (“split brain”) patients^9^. In that study, illusory contour or occlusion stimuli were placed in each hemifield. The left and right hemispheres of the patients were equally able to complete illusory contours; however, the right hemisphere preferentially supported occlusion. Moreover, electrophysiological recordings in macaque monkeys showed that neuronal activity in early visual areas V1 and V2 was elevated by illusory contours but diminished by occlusion^10^. However, there is also conflicting evidence that V1 and V2 respond to both illusory and occlusion stimuli^11^. Therefore, it remains unclear whether a separate or common neural mechanism mediates these different perceptual completion processes within early visual cortex.

Early visual areas may complete occluded portions of an object topographically and send this information to higher cortical regions for object recognition. Experiments in cats and macaque monkeys indicate that neurons in early visual regions support illusory contours, and that V2 activation is more integral than V1^8,11^. In humans, regional cerebral blood flow within V2 is increased whilst viewing illusory contour stimuli^5^. Optical imaging experiments suggested an inversion of orientation tuning for illusory contours in V1 but not V2: while tuning preferences for actual and illusory contours were correlated in V2, orientation tuning for illusory contours was orthogonal to that for actual contours in V1^3^. A more recent fMRI study demonstrated that the orientation of illusory contours is encoded across visual cortex, including V1^1^. These results converge to suggest that information about the contours themselves is present as early as V1.

Whilst these studies provide strong evidence for early visual cortex activation in response to illusory contours, they do not clarify whether this activation is due to feedback from higher areas. The lateral occipital complex (LOC) is located within extrastriate cortex and activated when participants view intact objects compared to scrambled versions^12–14^. An fMRI study found that illusory contours induce the strongest activation in a region encompassing high extrastriate cortex^15^. The LOC is also preferentially activated in fMRI studies on occlusion^16^. The LOC is situated higher in the visual processing hierarchy than V2. These results therefore advocate a “top-down” mechanism, in line with computational models emphasizing higher level surface construction and corresponding feedback^17^. While higher visual areas are preferentially activated by illusory contour stimuli, the LOC activation itself might reflect global completion processes that are independent of the illusory contour percept. Stimuli in which the sharp edges of the Kanizsa-type Pacmen are curved eliminate the perception of illusory contours but nonetheless induce an impression of an enclosed salient diamond-shaped region^7^. Comparable fMRI activation occurs in LOC for both stimulus conditions. Therefore, LOC activation may reflect a quick, crude surface construction or object recognition response rather than the phenomenal experience of illusory contours.

Further evidence for a multistage, recurrent process in the percept of illusory contours comes from transcranial magnetic stimulation (TMS) experiments^18^. While disrupting neural processing in LOC eliminates the illusory contour percept shortly after stimulus onset, TMS over early visual cortex does so at a later time. This suggests that feedback signals from LOC into early visual areas are required for the phenomenal experience of illusory contours to arise. This also concurs with the response properties of neurons in early visual cortex: V1/V2 neurons have small receptive fields^19,20^ that are selective for edge orientation^21,22^. This makes them ideally suited for assigning sharp object boundaries. Activity in these early regions might then be interpreted as sharp illusory edges. In contrast, LOC neurons respond to a wide area of the visual scene, frequently spreading over both hemifields^19,23^ and LOC is responsive to objects. It thus seems plausible that it extracts shape and surface properties and is only indirectly involved in detecting illusory contours.

To what extent occluded objects produce responses in early visual cortex remains less well understood. While V1 probably plays some role in spatial integration, it seems likely that higher visual areas, whose neurons have larger receptive fields and which are selective for more complex objects, mediate the processing of occluded objects. The behavioural deficits of a patient with atypical fMRI responses in V2 and V3, but normal responses in V1, support this interpretation^24^. However, a recent fMRI experiment combined a travelling wave design typically used for retinotopic mapping with occluded stimuli, and showed that occluded stimuli produce spatially selective signals as early as V1^2^.

Here we set out to use a similar design to test the presence of spatially selective signals in early visual cortex. We compared mapping stimuli defined by an occluded object with those defined by illusory Kanizsa-type contours and a subtle luminance stimulus that matched those conditions. Instead of a travelling wave design, we used bar stimuli that traverse the visual field in the four cardinal directions. Moreover, we employed pRF analysis which not only estimates the visual field position preferred by each voxel but also its spatial selectivity, the range of visual field locations where a stimulus can evoke a response, indicated by the size of the pRF^19^. We tested whether visual field maps estimated with these stimuli correlate with those estimated by real luminance contours and whether retinotopic selectivity (indicated by pRF size) and signal-to-noise ratios systematically differed between illusory and luminance stimuli. Further, we intended to reveal any systematic differences between areas V1–3 in encoding one or more of these stimulus types.

## Methods

### Participants

We obtained measurements from seven participants (five males and two females; age range; 22–36; all right handed) including both authors. All participants completed two fMRI experiments: The first used a combined wedges-and-ring stimulus to produce pRF maps. This mapping experiment was conducted as part of previous studies and details of the methods have been described elsewhere^25,26^. The second experiment used moving bar stimuli to measure the maps produced by illusory Kanizsa contours, occlusion and luminance-defined contours. All participants had normal or corrected-to-normal visual acuity. We acquired written informed consent from all participants in order to complete the different experiments and confirmation they understood the potential risks of fMRI. Procedures were carried out in accordance with the Declaration of Helsinki and were approved by the University College London Psychology and Language Sciences Ethics Committee.

### Stimuli

All stimuli were created using MATLAB (The Mathworks, Inc.) and the Psychophysics Toolbox (Brainard, 1997). The stimuli were projected (resolution 1920 × 1080 pixels) onto a screen (36.8 × 20.2 cm) at the rear of the scanner bore reflected off a mirror attached above the head coil. The viewing distance was 68 cm, resulting in a screen size of 30.1 × 16.9° of visual angle.

**Figure 2:**
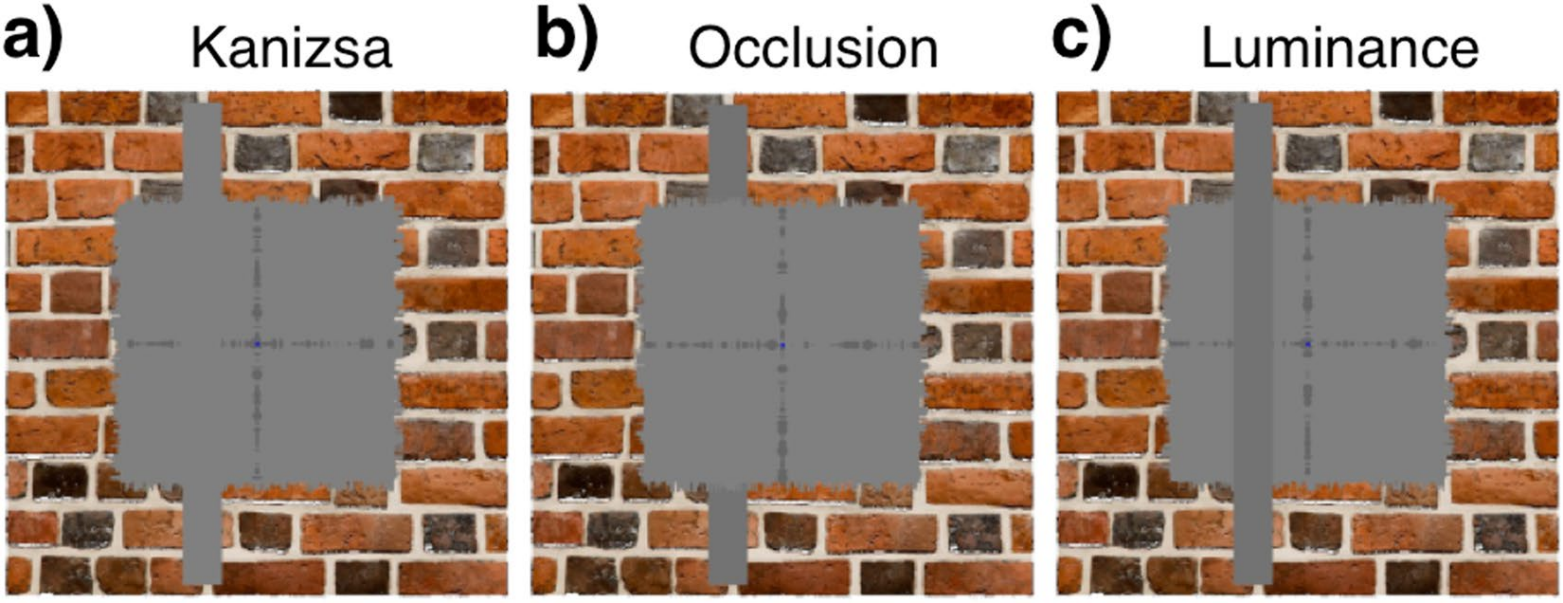
Stimulus conditions in this experiment. A bar stimulus traversed the region defined by the grey “carpet” occluding the brick image in four cardinal directions (up, down, left, right) stimulating a different position every second. (**a**) Kanizsa-type illusory contours. The colour of the bar stimulus and the carpet were identical resulting in a bar defined by illusory contours (the illusory contour percept was more pronounced with moving bars than in this static image). (**b**) Occlusion. The bar stimulus was slightly darker than the carpet but was presented behind it. (**c**) Luminance control. The bar stimulus was also darker than the carpet but was presented on top of it. All bars are shown with doubled (proportional) width for illustration purposes. For animated versions see stimulus GIFs available at https://osf.io/jmqkn/.

A background brick image of 728 × 728 pixels (11.4°) was displayed behind a square occluder of 428 × 428 pixels (6.7°). This occluder had the same grey colour as the screen background. The central fixation point of diameter 5 pixels (0.08°) was continually present. Similarly, two low contrast jagged lines creating a “plus sign” that covered the entirety of the inner grey square were presented throughout (Fig. 2). The brick image was taken from Wikimedia commons (goo.gl/aumdlN). In each condition the 10.9 × 0.9° bar overlapped with the brick image by 2.1° on either side. The bar moved in 30 equal discrete steps of 1 second in each of the four cardinal directions, flashing on and off at each step (2 Hz).

The display of the bar was manipulated in three experimental conditions: Kanizsa, Occlusion and the Luminance control conditions. In the Luminance condition, a grey bar was slightly darker than the grey background (pixel intensities: grey bar = 114; square occluder = 127). In the Kanizsa condition, the bar had the same colour as the grey background and the occluder. This meant that the 2.1 × 0.9° area in which each bar end would cover the brick image was the only part of the stimulus were any physical stimulation occurred, apart from the intersection of the bar with the plus sign (Fig. 2 and stimulus GIFs). For the Occlusion condition, the bar was filled with the same dark grey colour as in the Luminance condition, but the bar was presented behind the occluder – thus, there was no physical stimulation in the location of the occluder.

There was a 10 second period at the beginning of the run when only the background, brick border, fixation point and lines were visible. The volumes collected during that epoch were discarded to allow the fMRI signal to reach equilibrium. The bar was aligned either vertically or horizontally and traversed in opposing directions in distinct sweeps. A run contained six trials, four with the different bar directions/orientations and two null trials where no bar was presented. The order of the bar sweeps was: Right, Up, Left, Down. The null periods were always presented after the second and fourth sweep. Each trial lasted for 30 seconds. In total the runs were each 3 minutes and 10 seconds (6 × 30 seconds + 10 seconds of dummy scans at the start). All participants viewed either 12 runs per scan (4 for each condition) with the exception of one participant who viewed 9 runs (3 for each condition). The repeating order of the conditions (Kanizsa, Occlusion and Luminance) was varied across participants to control for order effects.

### Fixation tasks

The participants were instructed to continually focus on the fixation dot with a diameter of 0.08° and to complete a simple attention task. The fixation dot interchanged between blue and red colour. Participants watched out for red colour changes and tapped their right leg when this occurred. These attentional taps were unrecorded. The probability of the blue dot changing colour was 0.005 for every frame and red colour target epochs always lasted 200 ms. An eye tracker was used to monitor eye movements. (Eyelink 1000, sampling rate 60 Hz, 225 seconds per run). To determine gaze stability, we calculated the median absolute deviation of sampled gaze positions along the horizontal and vertical dimension for each run. We then averaged this measure for each participant and condition and compared the resulting indices of gaze stability between conditions using a repeated measures general linear model.

### Data acquisition

Functional and anatomical scans were acquired using a Siemens Avanto 1.5 T MRI scanner with a 32-channel Siemens head coil. The front attachment of the head coil was removed, therefore only 20 effective channels remained. Functional T2*- weighted multiband 2 D echo-planar images were taken with a multi-band^27^ sequence (TR = 1 ms, TE = 55 ms, 36 slices, flip angle = 75°, acceleration = 4) with a resolution of 2.3 mm isotropic voxels. A T1-weighted anatomical magnetization-prepared rapid acquisition with gradient echo (MPRAGE) image was acquired (TR = 2730 ms, TE = 3.57 ms) with a resolution of 1 mm isotropic voxels.

### Parameter estimation

Functional images were mean-bias corrected using custom software, then realigned and un-warped, and finally co-registered to the structural scan with SPM8 (http://www.fil.ion.ucl.ac.uk/spm). All further analysis was conducted using a custom MATLAB toolbox for pRF analysis (10.6084/m9.figshare.1344765.v22). The time series for each voxel in every run was linearly de-trended, normalized and averaged across runs. Data were then projected on a three-dimensional reconstruction of the grey white matter surface created by FreeSurfer^28,29^. This was achieved by finding the median position for each vertex of the surface reconstruction between the pial and grey-white matter boundary. The pRF analysis focused exclusively on the occipital lobe.

### Analysis

The procedure used for pRF modelling has been described in detail elsewhere^19,25,26,30^. In short, we used the overlap of a binary aperture describing the position of the (physical or illusory) mapping stimuli within each scanning volume with a profile of a pRF to predict the fMRI time series in the experiment. A coarse-to-fine optimization approach then determined the optimal pRF parameters for which the goodness-of-fit of the predicted time series to the observed data was maximized.

Visual areas V1, V2, V3, V4, and V3A were delineated based on reversals in the polar angle map derived from a standard mapping experiment, details of which can be found in van Dijk *et al*.^26^. In short, this experiment comprised two scanning sessions totalling 12 runs and used various high-contrast natural images which filled a ring and wedge aperture. The maximal eccentricity of this stimulus was 8.5° of visual angle. The bar stimuli within the main experiment reported here had a maximal eccentricity of 5.7° of visual angle. However, to exclude the response to the physical visual stimulations of the fixation dot and the outer brick border the pRF analysis was confined to the “carpet” (named for its fuzzy edges) region of interest falling within the grey occluder, excluding intersections with the jagged lines of the plus sign at the cardinal axes (Fig. 2). To define this rectangular region of interest, we restricted our analyses to pRFs with centre positions falling within 0.75° to 3.0° of visual angle from either cardinal axis in V1–V3, as determined in the standard mapping experiment. This way we restricted our analysis to four rectangular regions of interest in the visual field, each covering the area of a quadrant that fell within the carpet, but did not intersect with either the jagged plus sign or the edges of the carpet. Note that the cardinal axes were only physically stimulated in the illusory contours condition, but not the amodal completion condition. Nevertheless, we opted for this conservative criterion for all conditions, to keep the neural populations tested constant. We also did not include results from V4 and V3A in this report because larger pRF sizes in those regions would have led to substantial overlap of pRFs with the fringe region outside the “carpet” region where there was a physical difference in visual stimulation.

The visual field location of each pRF, its pRF size, and signal amplitude were calculated for all conditions. To test the consistency of these maps, we first correlated the observed time-courses in each condition with the time-course predicted for pRF parameters obtained in the mapping experiment. To determine the statistical significance of these correlations we ran a permutation test for each visual region and participant. Specifically, we calculated the (Fisher z-converted) average of correlations across vertices and compared it to the distribution of such average correlations across 10,000 iterations in which time-course predictions were shuffled across vertices. Note that this does not merely test correlations between illusion-evoked time-courses and mapping-based predictions, but whether these correlations are vertex-specific – in other words, the coherence of visual field maps for illusion and physical mapping stimuli.

Furthermore, we determined the vertex-wise correlation for pRF parameters between conditions, separately for each visual area. For these correlations we used the non-parametric Spearman rank correlation coefficient, except for the polar angle data for which we used circular correlation as in our previous study on the reliability of pRF parameters^26^. To test whether the size of correlations varied as a factor of visual area, pRF parameters and condition pairings, we z-transformed correlation values and submitted them to a repeated measures general linear model (GLM) with factors Area, Parameter and Condition Pairing and post-hoc t-tests. Finally, we quantified the proportion of responsive vertices, mean absolute pRF size and goodness of fit for each condition and visual area. These absolute values were also compared using repeated measure GLMS and post-hoc t-tests. General linear models were computed using JASP 0.8 (https://jasp-stats.org).

### Data availability

Data and code to reproduce results, as well as stimulus GIFs, are available at https://osf.io/jmqkn/.

## Results

### Gaze stability

**Figure 3:**
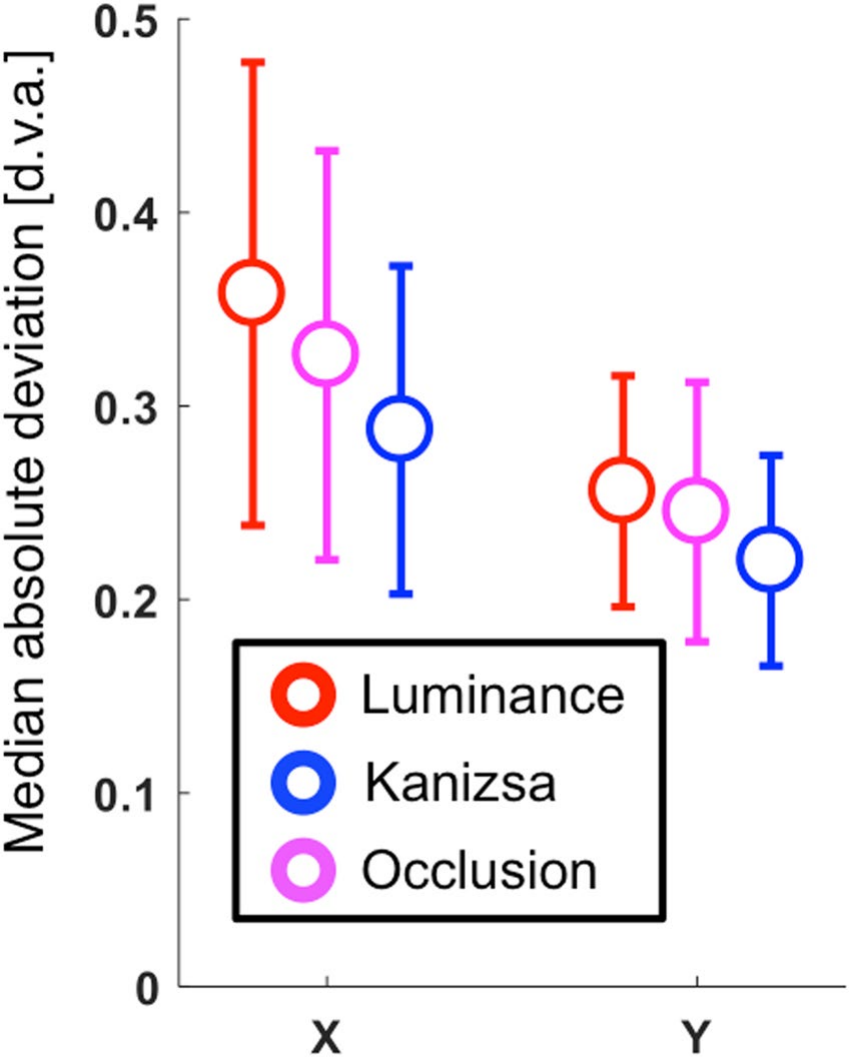
Gaze stability. To test observer compliance with the fixation instruction we calculated the run-wise median absolute deviation of sampled gaze positions in degrees visual angle (d.v.a.), separately for the horizontal (X) and vertical (Y) dimension. Data points and error bars show the mean + /− one standard error of the mean across observers for this measure. Colours indicate condition, as shown in the inset.

Eyetracking data indicated that participants were highly compliant with the fixation instruction. The run-wise median absolute deviation of gaze positions was below 0.5 degrees visual angle along the vertical and horizontal dimensions for all conditions (Fig. 3). Furthermore, a repeated measures general linear model showed no significant evidence for systematic differences between conditions with regard to this index of gaze stability (*F*_2,12_ = 2.16; *p* = 0.16; *η*^2^ = 0.27; and *F*_2,12_ = 1.52; *p* = 0.26; *η*^2^ = 0.20 for the horizontal and vertical dimensions, respectively).

### Correlation of time courses with predictions

The first step we took to test the coherence of spatial tuning across conditions was to correlate the observed time-courses for each condition with the predicted time-courses, based on pRF parameters obtained in the mapping experiment. We averaged these correlations across vertices for each visual area and participant and tested them against chance level using a permutation test that swapped predictions across vertices (see Methods, above).

**Figure 4:**
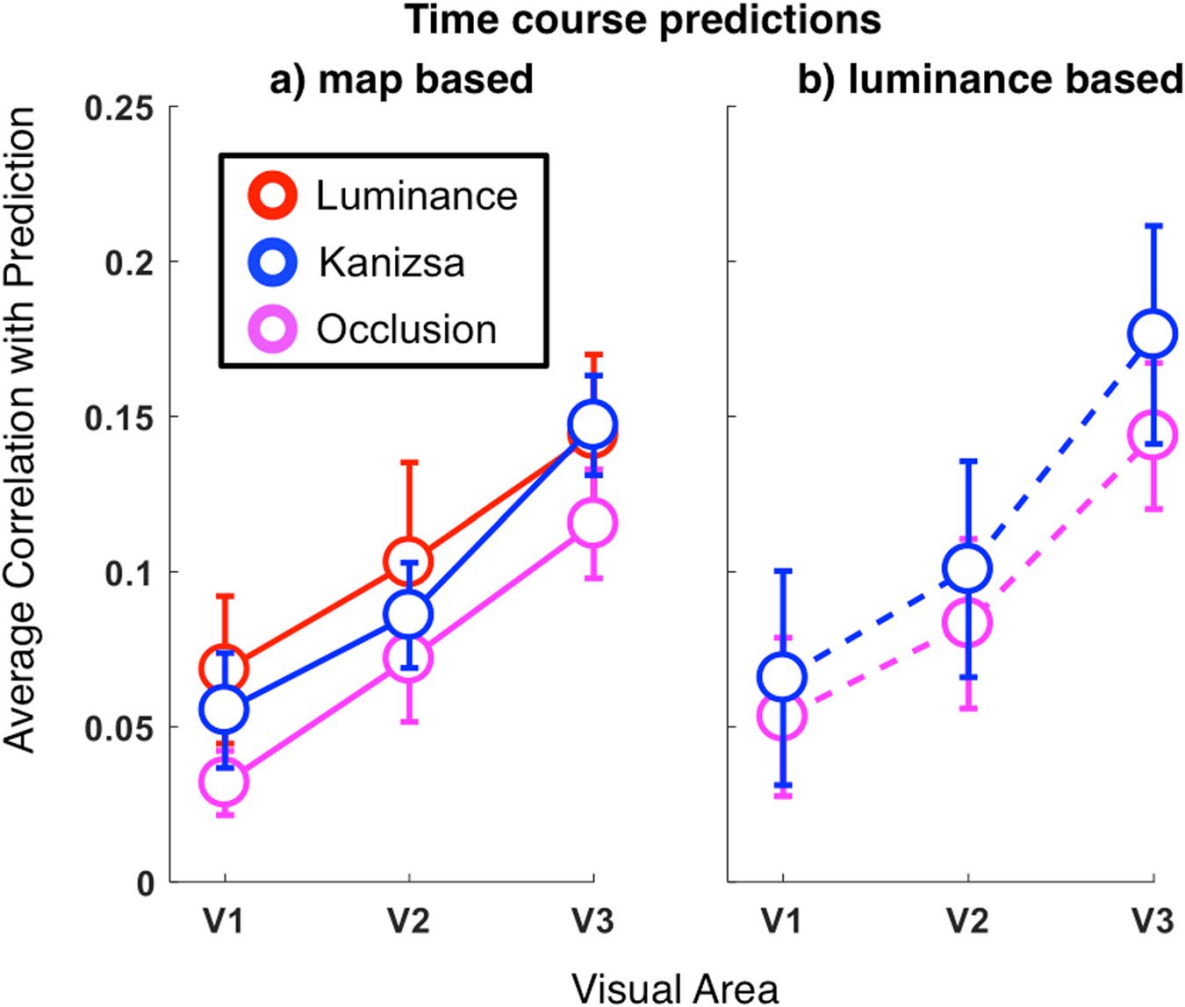
Correlations between observed time-courses and predictions based on (**a**) original map parameters and (**b**) parameters estimated in the luminance condition, for each visual area and condition. There was a significant increase in correlations from V1 to V2 to V3. Visual area and condition are indicated on the x-axis and by colour as shown in the inset, respectively. The y-axis shows average Pearson correlation coefficients. Averages were first computed across vertices for each participant and then across participants (both using Fisher Z-conversion). Circles and error bars show mean correlations + /− one standard error of the mean (S.E.M.) across observers. See main text and Methods for more details.

Figure 4a shows the resulting average correlations for each visual area and condition, in turn averaged across participants. Time-course correlations with predictions were very low overall, but the coherence of maps was preserved well above chance level (as indicated by permutation tests; p < 0.01 for at least 5 out of 7 participants for all visual areas and conditions apart from the Kanizsa condition in V1). Additionally, we observed a systematic increase of correlations across the visual hierarchy (from V1 to V3). A repeated measures general linear model showed this main effect to be statistically significant (*F*_2,12_ = 27.85; *p* < 0.001; *η*^2^ = 0.82; no other significant main or interaction effects). Post-hoc *t*-tests confirmed that correlations in all visual areas (across conditions) and all conditions (across visual areas) were significantly greater than zero (all *t*_20_ > 4.88; all *p* < 10^−4^; all Cohen’s *d* > 1.06) and increased from V1 to V2 to V3 (all pairwise *t*_20_ > 4.84; all *p* < 10^−4^; all Cohen’s *d* > 1.05).

This pattern was identical for time course correlations from both illusory conditions with predictions based on pRF parameters obtained in the luminance control condition (Fig. 4b). Here too, a repeated measures general linear model showed a significant main effect of visual area (*F*_2,12_ = 44.49; *p* < 0.001; *η*^2^ = 0.88), but no other significant main or interaction effects. Additionally, post-hoc *t*-tests confirmed that correlations in all visual areas (across conditions) and both conditions (across visual areas) were significantly greater than zero (*t*_13_ > 2.89; *p* < 0.05; Cohen’s *d* > 0.77 for all visual areas; *t*_20_ > 5.29; *p* < 10^−4^; Cohen’s *d* > 1.15 for both conditions) and increased from V1 to V2 to V3 (all pairwise *t*_13_ > 4.08; all *p* < 0.01; all Cohen’s *d* > 1.09). Finally, a 2 × 2 × 3 repeated measures general linear model across prediction type, illusion condition and visual area showed no significant main effect of or interaction with prediction type (map based or luminance based).

### Correlations between maps

**Figure 5:**
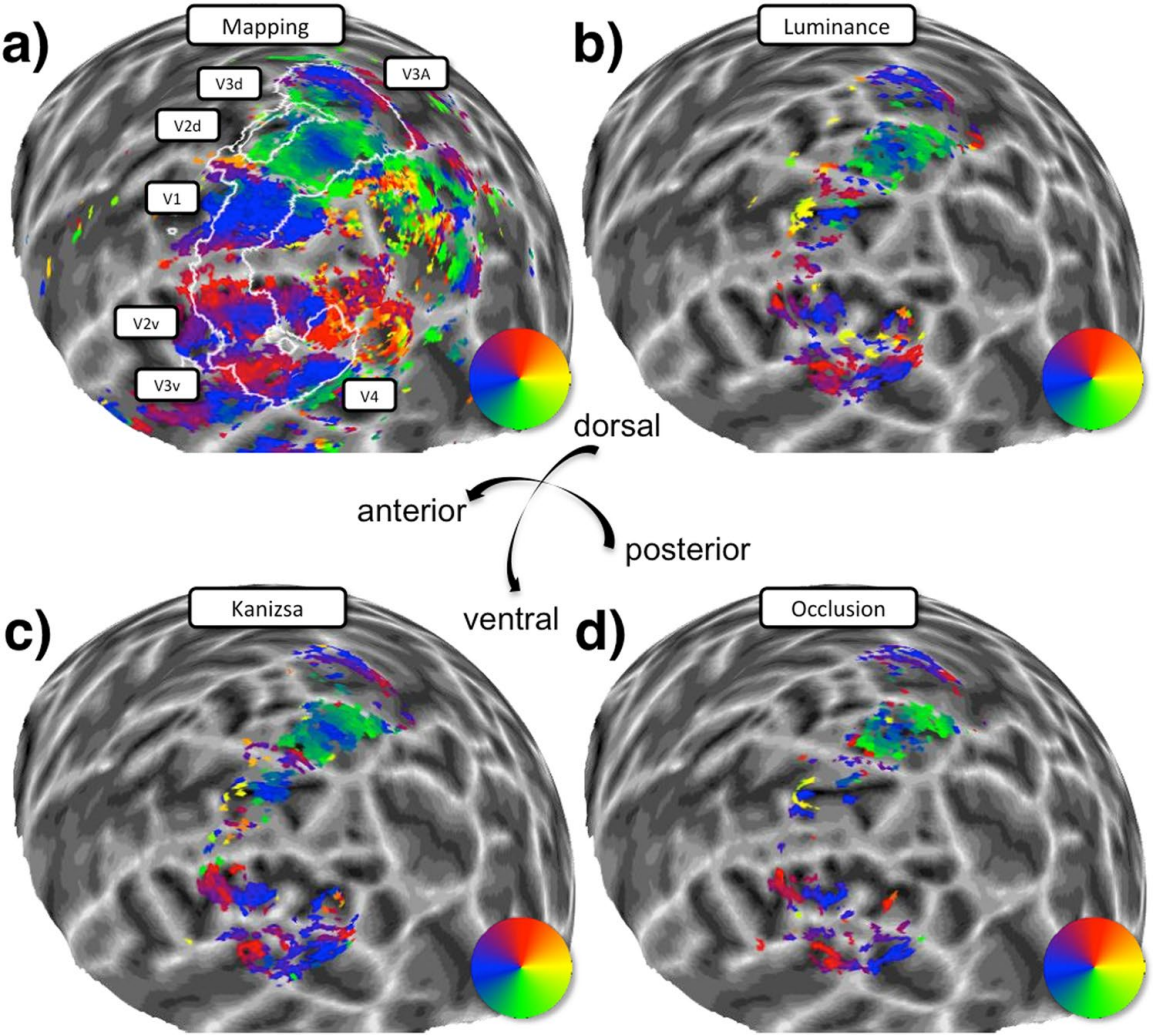
Sphere projection of polar angle data for an example hemisphere. The colour of each vertex indicates the best fitting polar angle parameter for the corresponding pRF centre (as indicated by the colour wheel). (**a**) Data from the independent mapping experiment served to delineate visual areas and regions of interest. The superimposed white outline corresponds to the area falling within the ‘carpet’ occluder (c.f. Fig. 2) and all other panels show data for this area only. Note that analyses were further restricted to data from V1–3 and pRF centres at a distance > 0.75 degrees visual angle from the cardinal ‘plus sign’ and the edges of the ‘carpet’ (c.f. Methods and Fig. 2). The remaining three panels show corresponding polar angle estimates for the low-contrast luminance (**b**), illusory contours (**c**) and occlusion (**d**) conditions.

**Figure 6:**
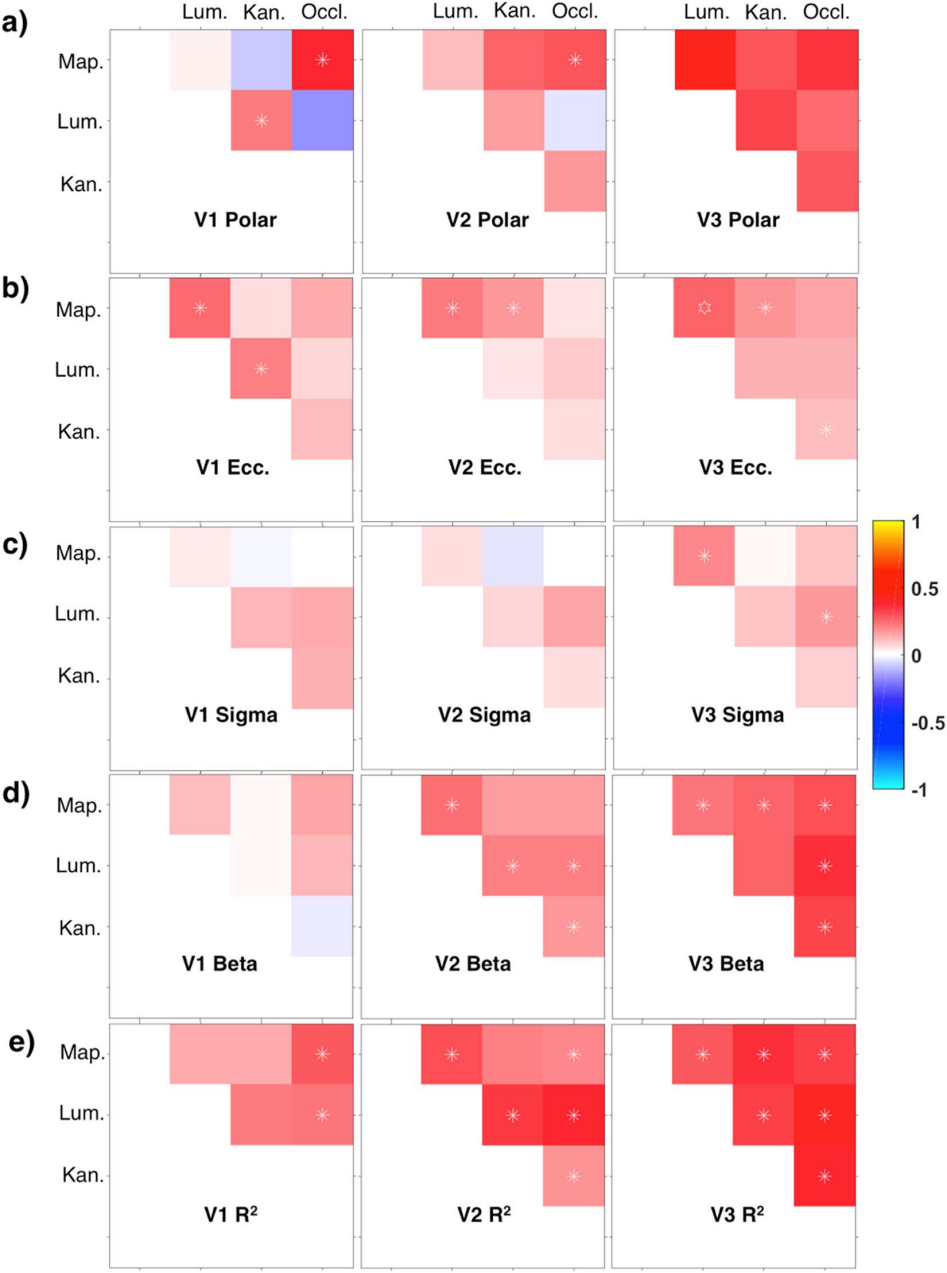
Correlation matrices comparing pRF position parameters between stimulus conditions for areas V1–V3. The colour of each cell denotes the strength and sign (see colour legend) of the vertex-wise correlation in polar angle (**a**), eccentricity (**b**), pRF size (**c**), response amplitude (**d**) and goodness of model fit (**e**). Correlations were calculated for each participant, then z-transformed, and averaged across participants. See Results for main (family-wise) statistical analyses. Symbols shown here indicate whether the average correlation in individual cells was significantly different from zero (uncorrected). Asterisks: p < 0.05. Star: p < 0.001. Map.: Mapping, Lum.: Luminance, Kan.: Kanizsa, Occl.: Occlusion.

In order to further quantify the similarity of maps between the different stimulus conditions we calculated vertex-wise correlations between them for the parameters fitted independently in either condition (see Fig. 5 for example maps). This analysis was performed separately for each visual region based on delineations made using the polar angle maps from the standard mapping experiment. Thus, we produced correlation matrices for areas V1–V3 comparing the patterns of each stimulus parameter in each of the four stimulus conditions: Mapping, Kanizsa, Occlusion, and Luminance (Fig. 6). The correlation coefficients were z-transformed and averaged across all participants.

This correlation analysis confirmed moderate consistency of polar angle estimates between stimulus conditions across all visual areas we tested, especially in V2 and V3. Patterns for eccentricity were less reliable and pRF sizes were only weakly correlated between illusory and luminance stimuli. Finally, response amplitudes and goodness of fit were positively correlated across most conditions, especially in V2 and V3. This indicates that vertices which responded more congruently with a retinotopic model for luminance stimuli, also had more reliable retinotopic responses for illusion stimuli.

A repeated measures general linear model confirmed significant main effects for visual area (*F*_2,12_ = 5.30; *p* < 0.05; *η*^2^ = 0.47) and pRF parameter (*F*_4,24_ = 4.91; *p* < 0.01; *η*^2^ = 0.45) but showed no significant main effect for condition pairing (*F*_5,30_ = 0.91; *p* = 0.49; *η*^2^ = 0.13). Post-hoc *t*-tests showed that correlations for each visual area (across condition pairings and parameters) were significantly greater than zero (all *t*_125_ > 5.77; all p < 10^−7^; all Cohen’s *d* > 0.51) and confirmed that correlations were increasing from V1 to V2 to V3 (all pairwise comparisons *t*_125_ > 2.59; all *p* < 0.05; all Cohen’s *d* > 0.23). They further showed that correlations for each parameter (across condition pairings and visual areas) were significantly greater than zero (all *t*_125_ > 4.51; all *p* < 10^−4^; all Cohen’s *d* > 0.40), that correlations for the R^2^ parameter were significantly greater than for all other parameters (all *t*_125_ > 2.22; all p < 0.05; all Cohen’s *d* > 0.19) and that correlations for the pRF size parameter (Sigma) were smaller than for all other parameters (all *t*_125_ < −2.78; all *p* < 0.01; all Cohen’s *d* < −0.24).

### Stimulus dependence of pRF parameters

Our correlation analysis tested the consistency of pRF maps for the different stimuli. In addition, we wanted to test whether condition-dependent differences in pRF size estimates were systematic and whether there was an offset in signal-to-noise ratios between conditions.

**Figure 7:**
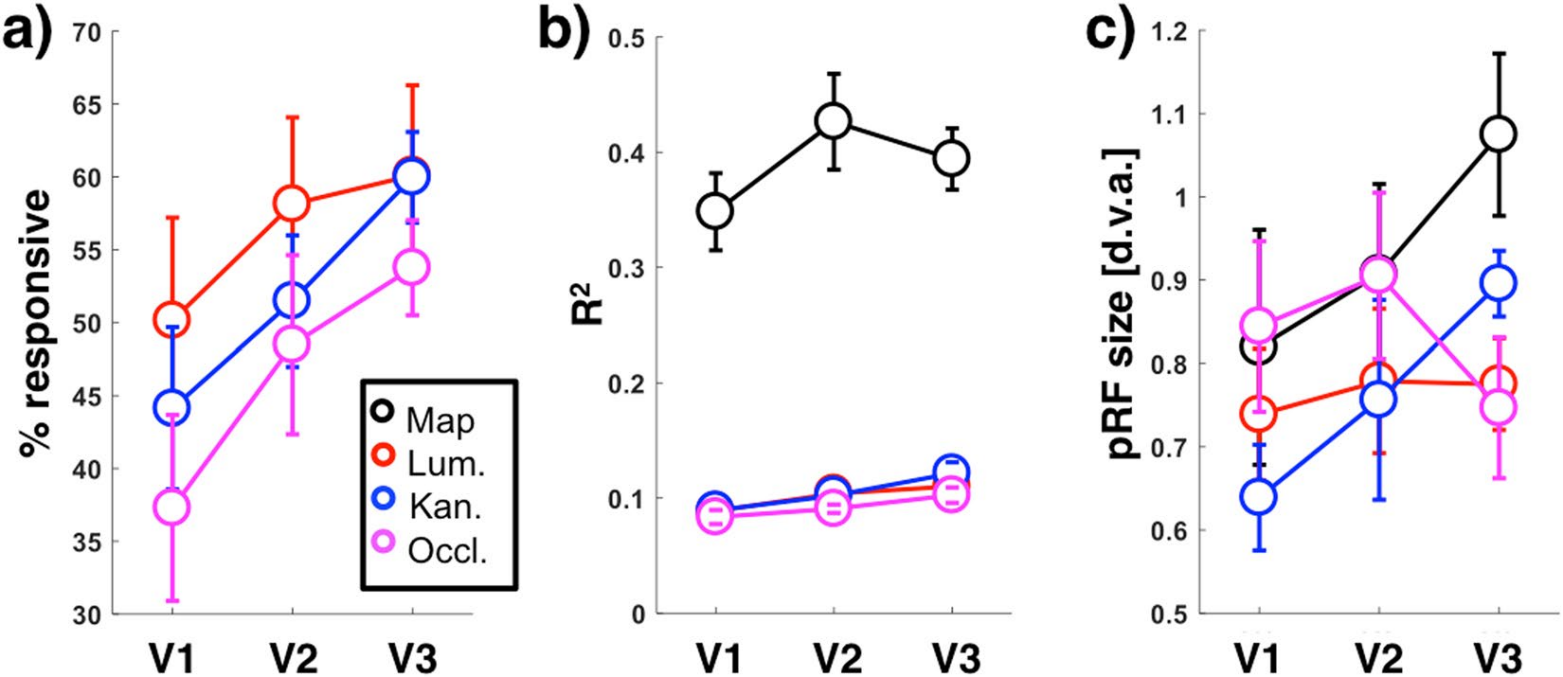
Proportion of vertices responding (**a**), goodness-of-fit (**b**) and pRF sizes in V1-V3 (x-axes), plotted for the different stimulus conditions. Circles and error bars denote mean values across participants + /− 1 standard error of the mean. Black: standard Mapping experiment. Red: Luminance control stimuli. Blue: Illusory Kanizsa stimuli. Purple: Occlusion stimuli.

For each participant and visual area, we first identified vertices with pRFs falling within the parafoveal area of illusory ‘stimulation’, as determined using the original mapping data. We then determined the proportion of these vertices responding retinotopically in the three conditions of interest (goodness of pRF model fit R^2^ > 0.05). This proportion ranged from 36–59% (Fig. 7a) and increased across the visual hierarchy (*F*_2,12_ = 4.05; *p* < 0.05; *η*^2^ = 0.40; post-hoc *t*-tests confirming higher proportions of vertices responding in V2 and V3 compared to V1; *t*_6_ > 3.56, *p* < 0.01; Cohen’s *d* > 1.34). However, there was no significant main effect of conditions (*F*_2,12_ = 1.50; *p* = 0.26; *η*^2^ = 0.47) or interaction effect (*F*_4,24_ = 0.90; *p* = 0.48; *η*^2^ = 0.13).

Comparing the goodness of fit across conditions (Fig. 7b) similarly showed an increase across visual areas (*F*_2,12_ = 4.61; *p* < 0.05; *η*^2^ = 0.44; higher R^2^ values in V2 and V3 compared to V1; *t*_27_ > 2.96, *p* < 0.01; Cohen’s *d* > 0.55). There was also a strong effect of condition, with R^2^ values in the original map (unsurprisingly) far exceeding those of the experimental conditions (*F*_3,18_ = 109.08; *p* = 0.001; *η*^2^ = 0.95; Map advantage over all other conditions *t*_20_ > 13.98, *p* < 0.001; Cohen’s *d* > 3.05). Goodness-of-fit was also slightly higher in the Kanizsa condition compared to the Occlusion condition (*t*_20_ = 3.01, *p* < 0.01; Cohen’s *d* = 0.66). Notably, there was no significant difference between goodness-of-fit between the two illusion conditions compared to the low-contrast luminance control condition.

Finally, parafoveal pRF sizes (Fig. 7c) were similar across all conditions, including original map data (*F*_3,18_ = 1.05; *p* = 0.49; *η*^2^ = 0.15), as well as across visual areas (*F*_2,12_ = 2.24; *p* = 0.15; *η*^2^ = 0.27). However, there was a significant interaction effect (*F*_6,36_ = 3.09; *p* < 0.05; *η*^2^ = 0.34) and post-hoc *t*-tests showed that original map pRFs in V3 were significantly larger than V1 pRFs in all conditions (*t*_6_ > 3.18, *p* < 0.05; Cohen’s *d* > 1.20) and original map pRFs in V2 (*t*_6_ = 3.11, *p* < 0.05; Cohen’s *d* = 1.18). Furthermore, V3 pRFs in the illusory contours condition were significantly larger than V1 pRFs in the same condition (*t*_6_ = 5.07, *p* < 0.01; Cohen’s *d* = 1.92).

## Discussion

Occlusion detection is a crucial ability of the human visual system that enables the connection of fragmented visual objects to produce a complete representation of the visual world^2^. Our study provides human neuroimaging evidence for retinotopic responses in early visual field maps in response to stimuli implying Occlusion (amodal completion), illusory Kanizsa contours (modal completion), and a Luminance control condition that was closely matched to the illusory stimuli but contained a low-contrast physical edge.

We observed that while the signal-to-noise ratio for our bar stimuli were unsurprisingly weak, all stimulus conditions elicited retinotopic responses in V1–3. The proportion of vertices responding, goodness of model-fit and coherence with independent mapping data systematically increased from V1 to V2 to V3. This was true for both illusory conditions, as well as for the low-contrast luminance stimulus. Moreover, there was no main effect of stimulus condition on map coherence. Model-fits were very low overall and slightly better in the illusory contours condition compared to the occlusion condition. However, importantly, neither illusory condition was significantly different from the low-contrast luminance condition. pRF sizes were overall very similar to those obtained for independent mapping data, but slightly increased across the visual hierarchy for mapping and Kanizsa stimuli, while this effect was not observed for the occlusion and low-contrast luminance stimuli.

Taken together, these findings suggest that both occlusion and illusory contour stimuli can be used to measure positional preferences of individual voxels, albeit with greatly reduced signal-to-noise ratios. Our findings are therefore a conceptual replication of previous neuroimaging and physiological experiments suggesting that occlusion and illusory contours elicit similar responses in early visual cortex^2,11^. Note that this would not preclude the possibility of differential upstream mechanisms driving these responses (e.g. in LOC, see below).

The fact that all experimental conditions produced poorer model fits is unsurprising given the considerable physical differences between these stimuli and the standard mapping stimuli. The latter contained high-contrast, coloured, natural images whilst the former were defined either by a subtle edge percept (Luminance and Kanizsa stimuli), or by no edge percept at all (Occlusion). However, we must also note that we collected considerably more data in the standard mapping experiment (12 runs of pRF mapping split across two scanning sessions) than we used for the experimental stimuli in the present study. Moreover, the mapping experiment employed a combined wedge-and-ring design that can produce better model fits and smaller pRF size estimates even when compared to bar stimuli that comprise high contrast stimuli and equivalent amounts of data^31^.

There is a strong possibility that the positional preference of voxels we identified may correspond to spatial attention to the location of the bar (or its inferred location) rather than specific perceptual qualities of the stimuli. Despite parameter correlations between conditions being very low (esp. in V1; c.f. Fig. 6), the overall pattern of results was highly similar across conditions. All conditions produced fits with low signal to noise levels that improved across V1–3 (Fig. 7a,b). For all conditions, this increase in model fits went along with an increase in between-condition parameter correlations (c.f. Fig. 6) and an increase in correlations with time-course predictions based on physical stimuli (Fig. 4). This suggests that the limited nature of parameter correlations is likely due to the (unsurprisingly) high noise levels in the experimental conditions, rather than any systematic differences between them. Given these similarities, a common mechanism behind the retinotopic activations we observed seems possible (again, please note that this would not preclude the possibility of differential upstream mechanisms supporting either percept). We thus hypothesize that covert spatial attention is responsible for the visual field maps in our experiment. This concurs with previous findings that the topographical organization of visual cortex is activated by spatial attention. It is a well established finding that spatial attention can drive responses in early retinotopic cortex (e.g. refs^2,32–35^) and retinotopic maps can be measured entirely by modulating spatial attention^36,37^. Compared to parietal and frontal regions, maps derived by attentional modulation were sparser in early visual areas but nevertheless clear enough to generate retinotopic maps. Previous studies^33,36,38^ also found an increase of attentional modulation effects along from V1 to V3, further matching the pattern of results we observed here.

Ban and colleagues^2^ used a similar design to ours and showed that an occluded wedge stimulus can generate reliable polar angle maps in early visual cortex (using phase-encoded retinotopic mapping). Our results from the Occlusion stimuli corroborate their conclusions. However, given the similarity between the retinotopic maps between all our experimental conditions, we propose that this may also reflect the effect of spatial attention. Ban and colleagues sought to control for this by showing that it was still possible to measure these maps while participants performed a demanding fixation task that should have withdrawn attention from the mapping stimulus.

However, attentional deployment is not binary^39^ and thus a demanding task at fixation does not necessarily rule out spatial deployment of attention in other locations of the visual field. In fact, withdrawing attention from a retinotopic-mapping stimulus using a demanding task at fixation results in modulation of pRF parameters in early visual cortex, specifically an increase in eccentricity estimates and pRF size^40^. Moreover, withdrawing attention sufficiently from a Kanizsa or occlusion stimulus may disrupt the percept of illusory contours or the awareness of an occluded object. Therefore, it becomes impossible to disentangle whether or not any neural correlates of the stimulus are due to attentional deployment: if a demanding attention task obliterated the maps we measure with Kanizsa or occlusion stimuli, this could simply be due to the fact that a minimum level of attention is required to perceive such stimuli in the first place.

In summary, our study revealed few systematic differences between the retinotopic responses to illusory contours, occlusion and subtle luminance stimuli in early visual cortex. One such difference was a slight increase in pRF sizes across the visual hierarchy for mapping and Kanizsa stimuli, which was not observed for the occlusion and low-contrast luminance conditions. Given the low signal to noise levels, our limited sample size and general limitations of fMRI, we cannot rule out the existence of further subtle, but systematic differences, for which our methods may have lacked sensitivity. Furthermore, even if illusory contours and occlusion go along with similar activations in V1–3, their percept likely is supported by different neural mechanisms, as suggested by previous findings^9^. Further research is required to explain the perceptual discrepancies between illusory contours and occlusion. An electroencephalography (EEG) study comparing completion processes found comparable activation within the LOC and parietal structures at 140 ms subsequent to the initial stimuli onset, but a later dissociation in the activation strength at 240 ms within the higher cortical areas^41^. Thus, differences in the processing of Kanizsa and occlusion percepts may occur late after stimulus onset and/or involve higher cortical areas.

This hints at a practical limitation of our study: the temporal resolution of fMRI is considerably poorer than that of electrophysiological measures. Further, fMRI measures the blood oxygenation level dependent signal rather than directly measuring neuronal responses^42,43^. Therefore, we cannot rule out that the different stimuli are processed differently at the neuronal level but this is obscured by the indirectness of hemodynamic responses.

Our study also has low statistical power for detecting weak effect sizes and this reduces the probability of detecting subtle differences in pRF parameters. We clearly observed coarse differences between our standard mapping experiments and our moving bar stimuli in the present experiment. Naturally, however, with our sample of 7 participants we are unable to rule out subtle differences between the Kanizsa, Occlusion and Luminance conditions. Importantly, we did not set out to test such subtle effects. Rather, we sought to establish whether any signals in response to these stimuli could be detected and used for retinotopic mapping *in principle*. This was clearly the case. The fact that consistent pRF position estimates could be obtained for all three stimuli suggests that these signals are unspecific to the phenomenon of occlusion or illusory contours but instead reflect more general processing, such as spatially selective attention.

## Conclusion

We found retinotopic responses for illusory Kanizsa contours, occluded bar stimuli and luminance control stimuli. These responses had low signal-to-noise ratios, but were significantly correlated with independent high-contrast mapping data and corresponding model-predictions. Furthermore, we found no differences in the proportion of vertices responding and signal-to-noise ratios between illusion and low-contrast luminance stimuli. Therefore, we propose the retinotopic activation produced by occlusion and illusory contours stems from a general process shared by all stimuli, rather than being directly related to the perceptual qualities specific to each condition. One candidate for such a general process is spatially selective attention to the inferred bar location.
